# Martha Rogers’ science of unitary human beings in relation to workers health and well-being: A scoping review

**DOI:** 10.3233/WOR-220681

**Published:** 2023-11-10

**Authors:** Åsa Hedlund

**Affiliations:** Department of Caring Sciences, Faculty of Health and Occupational Studies, University of Gävle, Gävle, Sweden

**Keywords:** Nursing research, nursing theory, occupational groups, occupational health

## Abstract

**BACKGROUND:**

Workers’ health and well-being are topics on the rise within occupational research. Rogers’ science of unitary human beings can potentially contribute to increased knowledge in the area. However, no previous review has investigated how the theory has been used in relation to workers in working life.

**OBJECTIVE:**

The aim of this scoping review was to provide an overview of studies that have used Rogers’ science of unitary human beings to study workers health and well-being in working life.

**METHODS:**

A literature search was conducted in CINAHL and PubMed, and other relevant sources in May-June 2022.

**RESULTS:**

The results showed that there seems to be a lack of use of Rogers’ science of unitary human beings regarding workers health and well-being in working life. The overarching theme was: Well-being as an essential phenomenon in working life in all dimensions of existing.

**CONCLUSION:**

The theory has potential to contribute more to research regarding workers’ health and well-being in working life.

## 1 Introduction

Workers’ health and well-being at work are growing topics in contemporary research, but more knowledge is needed. The contents of and interactions in working life are crucial for workers’ health and well-being [1–4]. Martha Rogers’ science of unitary human beings assumes that individuals are evolving beings, integral with their environment, which affects their health and well-being [5]. As yet, no review has investigated how Rogers’ theory has been studied among workers, and what potential the theory has to increase knowledge in working life research.

Working life is defined as the part of an individual’s life when they “do a job or are at work” [6]. This involves for example having daily routines, regular income [1], and opportunities for development [3]. A significant part of working life is the work environment, which can be defined as the settings, situations, conditions, and circumstances under which people work, according to a conceptual review by Oludeyi [7]. This encompasses three aspects: the human aspect (i.e., other people), the technical aspect (e.g., equipment and technological infrastructure), and the organizational aspect (e.g., policies, roles, and philosophies) [7]. The nature of working life, the characteristics of the worker, and the interaction between the two are important for workers’ health and well-being. For example, pain and stress-related issues such as burnout are common health problems [8–10], and aspects of working life (e.g., high workload, working long hours, or bad ergonomics) as well as aspects within the individual (e.g., lack of experience, neuroticism) are important risk factors for them [10–12]. However, workplaces have or should have the potential to promote health and well-being among their employees. The European Network for Workplace Health Promotion [13] has exemplified this with involving employees in decision-making, organizing work tasks in a health-promoting way, and have a working culture based on partnership. The Centers for Disease Control and Prevention in the United States [14] has stated that, e.g., reducing noise at work, the level of air pollution and having safe equipment are important for workers’ health. A systematic review showed that it is possible to enhance health, well-being, and work outcomes among workers with interventions that target the workplace’s physical environment and organizational structure [15].

Though the importance of the nature of working life for workers’ health and well-being is recognized, nursing theory seems to be underused in this area. A potentially relevant nursing theory for research related to working life is Martha Rogers’ science of unitary human beings. This is a grand theory or conceptual model that has been applied in a broad range of areas and methodologies [16] since its introduction in 1970 [5]. According to the theory, humans are energy fields that are more than the sum of their biological, psychological, physiological, social, cultural, and spiritual parts, and outcomes cannot be predicted from the parts. The environment is also defined as an energy field and consists of everything external to the individual, encompassing the whole universe. The integrality between the individual and the environment creates a unitary energy field that becomes manifest in behaviors, health, and well-being (the two latter are usually used synonymously) [5]. This occurs in a creative, evolving, progressive, and rhythmical life process that moves towards greater complexification through space and time (pan-dimensionality) [5]. This energy field have a wave pattern that is dynamic and changes from moment to moment, and its traits are described based on openness and the three principles of homeodynamics: resonancy, helicy, and integrality [17]. The theory is optimistic and speaks about new visions, flexibility, curiosity, imagination, courage, risk-taking, compassion, and humor [17]. Several middle-range theories have been developed from this theory, such as the Health Empowerment Theory, Power as Knowing Participation in Change, and the Theory of Healthiness [18]. The theory is highly abstract but is nevertheless considered suitable for framing both qualitative and quantitative research [17]. An overview of the use of the theory in 2008 showed that the concepts used were often different aspects of health/well-being, such as depression, pain, and quality of life [16]. The theory was also often used in studies including some kind of integrative/complementary treatment, such as healing or therapeutic touch. A few studies were found to focus on workers, but most samples included only individuals with health problems [16]. It seems that no review regarding Rogers’ science of unitary human beings has been conducted regarding workers health and well-being in working life. Therefore, the aim of this review was to provide an overview of studies that have used Rogers’ science of unitary human beings to study workers health and well-being in working life. Research questions were:

1. How has the theory been used – assessed using Silva’s [19] classification?
2. What was the quality of the studies?
3. How has the theory-concept environment been used in relation to work environment?
4. What were the major findings of the studies?

## 2 Methods

### 2.1 Design

This review aimed to broadly map the literature regarding workers health and well-being in relation to the science of unitary human beings. This is an appropriate method to do to prepare for a more comprehensive review [20]. Reporting of the study followed the PRISMA extension for scoping reviews (PRISMA-ScR) checklist for reporting scoping reviews [21], where applicable.

### 2.2 Search methods

The database searches were performed with support from a specialized librarian, to decrease the risk of bias, and were inspired by recommendations from the Cochrane handbook [22], and the PEO framework (Population – Exposure – Outcome) [23]. To identify relevant search terms, initial searches were conducted in PubMed and CINAHL. The articles found were screened for index terms and words in title and abstract. The literature search was then performed in PubMed and CINAHL in May-June 2022. The journals Holistic Nursing Practice and Nursing Science Quarterly were also screened for relevant articles, as were all available issues of Visions: The Journal of Rogerian Scholar Science. Further, PROSPERO was screened for relevant protocols. The search process began with single searches with terms relating to workers and Rogers’ theory. Both free-text and subject headings were used when possible. Then, terms relating to the same concept (e.g., Rogers’ theory) were combined using the Boolean term “OR.” Thus, there was one search block relating to workers and one search block relating to Rogers’ theory. These blocks were then combined with the Boolean term “AND” to direct the search more towards the aim of this review. The search strategy is presented in detail in Tables 1, 2.

**Table 1:** Search strategy CINAHL 2022-06-08

Search number	Search terms	Records (n)
S1	Nurses	552 347
S2	(MH “Nurses”)	67 500
S3	Professionals	487 050
S4	Staff	175 094
S5	Employees	59 374
S6	(MH “Employees”)	560
S7	Personnel	250 927
S8	Workers	113 172
S9	S1 OR S2 OR S3 OR S4 OR S5 OR S6 OR S7 OR S8	1 236 837
S10	“Theory of unitary human beings”	10
S11	“Unitary human beings”	795
S12	“Rogers’ theory of unitary human beings”	4
S13	“Rogers’ science of unitary human beings”	769
S14	“Science of unitary human beings”	788
S15	“Rogerian science”	37
S16	Pandimensionality	6
S17	“Unitary human caring science”	2
S18	“Unitary human caring”	2
S19	“Pattern manifestations”	24
S20	“Human energy field”	44
S21	“Environmental energy field”	9
S22	Helicy	15
S23	Resonancy	10
S24	(MH “Rogers Science of Unitary Human Beings”)	750
S25	S10 OR S11 OR S12 OR S13 OR S14 OR S15 OR S16 OR S17 OR S18 OR S19 OR S20 OR S21 OR S22 OR S23 OR S24	841
**S26**	**S9 AND S25**	**304**

**Table 2:** Search strategy PubMed 2022-05-20

Search number	Search terms	Records (n)
#1	“Occupational groups” [MeSH]	685 380
#2	Nurses [All Fields]	413 483
#3	Nurses [MeSH]	95 321
#4	Professionals [All Fields]	171 700
#5	Staff [All Fields]	287 899
#6	Employees [All Fields]	824 106
#7	Personnel [All Fields]	866 562
#8	Workers [All Fields]	890 669
#9	#1 OR #2 OR #3 OR #4 OR #5 OR #6 OR #7 OR #8	1 631 330
#10	“Theory of unitary human beings” [All Fields]	5
#11	“Unitary human beings” [All Fields]	131
#12	“Rogers’ theory of unitary human beings”	2
#13	“Rogers’ science of unitary human beings”	2
#14	“Science of unitary human beings” [All Fields]	124
#15	“Rogerian science” [All Fields]	15
#16	Pandimensionality [All Fields]	4
#17	“Unitary human caring science” [All Fields]	1
#18	“Unitary human caring” [All Fields]	1
#19	“Pattern manifestations” [All Fields]	9
#20	“Human energy field” [All Fields]	18
#21	“Environmental energy field” [All Fields]	4
#22	Helicy [All Fields]	4
#23	Resonancy [All Fields]	4
#24	#10 OR #11 OR #12 OR #13 OR #14 OR #15 OR #16 OR #17 OR #18 OR #19 OR #20 OR #21 OR #22 OR #23	167
**#25**	**#9 AND #24**	**58**

### 2.3 Search outcomes and study selection

**Fig. 1:**
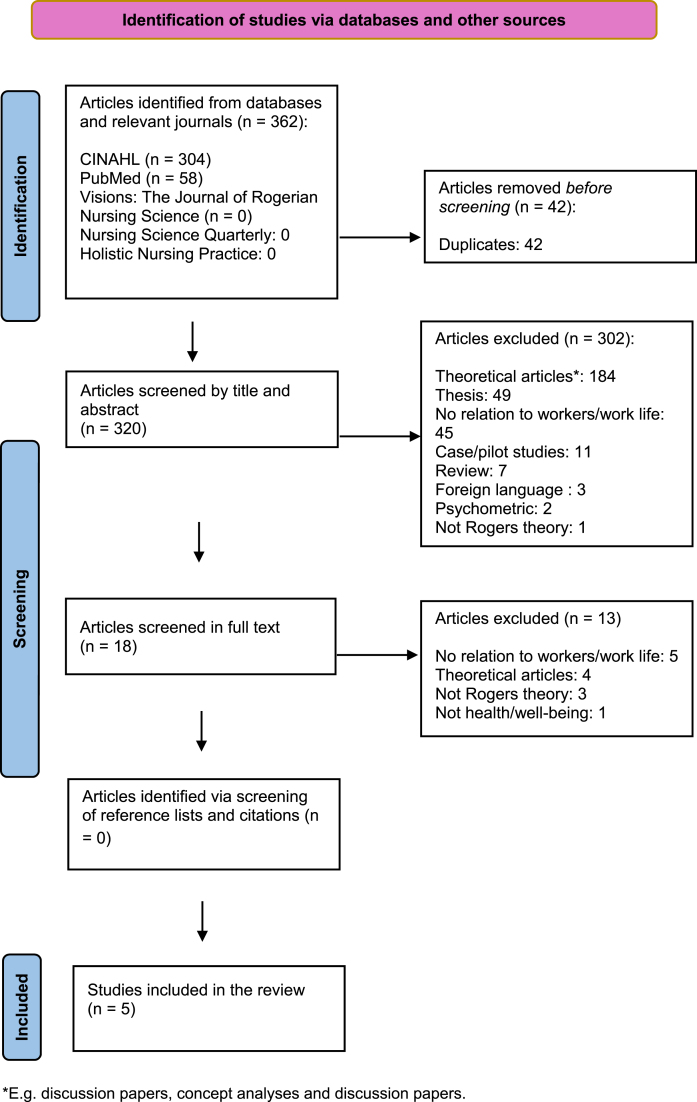
PRISMA 2020 Flow diagram illustrating the selection process.

The inclusion criteria were original studies with qualitative, quantitative, or mixed methods approach, regardless of design. The studies had to have used Rogers’ science of unitary human beings to study workers health and/or well-being in working life. Reviews, theoretical articles (e.g., concept analyses, editorials, columns and discussion papers), case studies, pilot studies, psychometric studies, studies only focusing on patient care, studies with no relation to working life (e.g. studies among workers well-being only in private life), theses, articles in other languages than English, and articles only including other theories than Rogers’ (e.g. middle-range theories derived from Rogers’ theory) were excluded. As the initial search indicated that there was a limited number of articles in the area, no timeframe was chosen. In the first step of the selection process, duplicates were removed. The remaining articles were then screened for eligibility based on the inclusion and exclusion criteria, in a step-wise fashion (see Fig. 1, PRISMA Flow diagram) [21]. Subsequently, the reference lists of the studies that met the inclusion criteria were screened, as well as the article citations in Scopus and Web of Science. However, no additional article was identified in this way. In total, five studies met the inclusion criteria.

### 2.4 Data analysis

The first research question was answered using Silva’s [19] classification of theory use, i.e., minimal use, insufficient use, or adequate use of theory. Minimal use means that a theory is briefly summarized in a study but does not really contribute to the research. Insufficient use means that a theory helped the researchers to organize their study, e.g., in choosing instruments for data collection. Adequate use is when a theory is explicitly tested, e.g., that study aims are derived from the theory. Silva described seven criteria for assessing the use of theory, which formed the basis for the assessment. The seven criteria were structured into a table, and the application of the theory in the included studies was analyzed and entered into the table. To answer research question 2, quality assessment of the included studies was performed using the Joanna Briggs Institute’s critical appraisal templates for cross-sectional and qualitative studies [24] and the Mixed Method Appraisal Tool (MMAT) for mixed-method studies [25]. No study was excluded due to quality limitations, as article quality is not the main focus in a review about use of theory [26]. Quality of study methodology and theoretical use are different research areas, meaning that high-quality empirical methods do not necessarily cooccur with adequate use of theories and vice versa [26]. However, quality assessment provides important information about the kind of studies in which a theory has been used. To answer research question 3, a manifest deductive content analysis [27] was performed, based on the aspects of work environment described in Oludeyi’s conceptual review [7]. First, a matrix including the three aspects of work environment was created, and data regarding work environment were extracted from the articles and compiled in a document. Then, the extracted data were interpreted based on if they belonged to the human aspect, the technical aspect, or the organizational aspect, and sorted into the matrix. To answer research question 4, a standardized extraction form, as described by Whittemore and Knafl [28] was used. This is a way to ensure that the same kinds of data were extracted from all studies, so they could be compared. Inspired by Whittemore and Knafl [28], the findings were extracted from the studies and entered into the form. Then, the data were compared across the studies to identify similarities, relationships and differences to create groups/categories, which then were abstracted to create an overall theme.

## 3 Results

### 3.1 Study characteristics

Of the five included studies, one was from 2016 [29], one from 2006 [30], one from 2005 [31], one from 1996 [32], and one from 1994 [33]. All studies originated from the United States, but had different authors. Four of the studies had a quantitative (cross-sectional) approach [30–33] and one used a mixed method approach, including a pretest-posttest design [29]. All study populations encompassed nurses. The studies related to working life in different ways, i.e. therapeutic touch at the work place with colleagues [29], nurses’ level of interaction with their nursing environment [30], job satisfaction [31], stress at work [30, 31], measures of work culture and work characteristics [33] and nurses’ social support at work and their willingness to care for AIDS-patients [32]. An overview of the study characteristics is provided in Table 3.

**Table 3:** Characteristics of the included studies

Authors, country, year	Aim	Design	Sample	Method for data collection	Data analysis	Major findings
Bulette Coakley, Barron & Donahue Annese, 2016, USA	To test the efficacy of a therapeutic touch (TT) intervention in influencing the level of stress, sense of comfort and well-being. Research question: What is the experience and impact of providing and receiving Therapeutic Touch treatments on nurses working in oncology and respiratory nursing?	Mixed method with pretest and posttest	Quantitative: A convenience sample of staff nurses on the Bone Marrow Transplant Unit and the Respiratory Acute Care Unit that were certified to perform TT and/or were willing to receive TT and discuss the experience Qualitative: Nurses who participated in the quantitative study were invited to the qualitative part.	Quantitative: Collection of quantitative measures of stress (e.g. heart rate), anxiety (The Spielberger State/Trait Anxiety questionnaire), comfort and overall well-being (Visual analogue scale) before and after TT-treatment. Qualitative: Focus group interviews.	Quantitative: Paired t-tests. Qualitative: Content analysis.	Quantitative: Recipients as well as providers of TT had significantly differences in the assessment of anxiety (decreased), comfort (increased), and well-being (increased). Also, nurses who received TT had significantly reduced respiratory rates. Qualitative: Nurses offering TT were moved to see that they helped colleagues to feel better, but it could be difficult to focus on providing the treatment because they were working on a busy unit and couldn’t stop thinking of everything they should do. Both recipients and providers described TT as valuable for promoting comfort, increase energy, relaxation, peacefulness, and promote a healing workplace/a healing experience. TT could also decrease pain and give a sense of meaning to do something good for others.
Lucero & Sousa, 2006 USA	To test the relationship between level of participation and change (an experience appraised by level of stress).	Quantitative, cross-sectional	75 nurses who worked in medical/surgical or intensive care units	The *Person-Environment Participation Scale* to measure the perceived level of interaction with the nursing environment. The *Perceived Stress Scale* as a proxy to measure change. Demographic questions such as age, gender, education level and length of employment.	Pearson product moment correlations, ANOVA, multivariate linear regression analyses.	Nurses were actively engaged in the nursing environment (i.e. not passive participants in it). They perceived stress sometimes to fairly often the last month. There were no differences in participation and stress between demographic groups. A higher level of engagement in the nursing environment was associated with lower levels of perceived stress (interpreted as degree of change). Regression analyses with participation (model 1) and stress (model 2) as the dependent variables and demographic as the independent variables showed that the variance accounted for was low (1,5% and 6,7% respectively) and that the demographics were not significant.
Hurley, 2005, USA	Unclear formulation but interpreted as: To quantitatively examine nurse managers experience of job satisfaction, stress, and power	Quantitative, cross-sectional	124 female managers (nurses)	The *Self-Anchoring Striving scale* to measure the managers perception of their stress experience and manifestations of stress, i.e. individualism, challenge, opportunity and consciousness raising. *The Knowing Participation* in Change Test to measure power. The *Work Quality Index* to measure job satisfaction. Demographic data, i.e. age, number of children, marital status, years in nursing, years in the current position, educational preparation, geographic location, size of institution, type of institution, size of unit managed, direct care responsibility, salary, overall life satisfaction, experience and amount of stress, experience of crisis and participation in fate were collected as well. Open-ended questions were asked regarding the managers perception of the experience of stress (not found in the results).	Pearson product moment correlations, t-test, analysis of variance, chi-square.	Overall, managers were satisfied with their jobs, i.e. 96.5% reported moderate or high job satisfaction. 52.5% rated the experience of stress medium and 44.2% rated it as high. Job satisfaction was not related to stress manifestations. Power was positively and significantly correlated with job satisfaction. Managers who had direct care responsibilities, had been in their position longer, had experienced a recent crisis had higher stress scores.
Rizzo, Gilman & Mersmann, 1994, USA	Unclear formulation but interpreted as: To investigate facilitation of care delivery redesign using measures of unit culture and work characteristics (The unit could, if they wished, use the results to integrate their units unique culture into a model development described in the article. A follow-up was going to be conducted.)	Quantitative, cross-sectional	Nurses from an oncological unit. Number unknown. They were a part of a larger sample with 235 nurses from a total of 13 units.	The *Nursing Unit Cultural Assessment Tool* where the nurses rated behaviors that they preferred versus behaviors that they believed occur typically on their workplace. The *Work Characteristics Instrument* for measure nurses perceptions of exciting, interesting and frustrating aspects of their work.	Pearson product moment correlations, means	Most important of the typical cultural behaviours to the nurses on the oncological unit were being competent, being comfortable in watching for life threatening complications and make patients comfortable. The frustrating aspects with work concerned staffing, communication and insufficient time to complete work tasks. Being respected for knowledge, have a stimulating environment, an interesting and exciting work and the opportunity to work with other professionals were the aspects of work that were strongest correlated to work excitement.
Sherman, 1996, USA	To examine relationships among spirituality, perceived social support, death anxiety, and nurses’ willingness to care for AIDS patients	Quantitative, cross-sectional	Female nurses from eight medical centers who provided care for patients with AIDS	The *Willingness to Care for AIDS Patients Instrument*, the *Spiritual Orientation Inventory,* the *Personal Resource Questionnaire-85*, the *Templer Death Anxiety Scale* and demographic questions.	Pearson product-moment correlations, hierarchical multiple regression analyses	The nurses had a relatively high death anxiety and moderately spirituality. Spirituality and perceived social support were positively associated with willingness to care for AIDS patients, while death anxiety was negatively correlated with it.

### 3.2 Theory use assessed using Silva’s classification

One of the studies [29] had minimal use of the theory. This meant that the theory was mentioned in the introduction as being related to the exposure in the study, without contributing more to the study. Two studies had insufficient use of the theory [30, 33], i.e. the theory guided data collection but was not tested per se. Two of the studies [31, 32] used the theory adequately, i.e., it shaped the hypotheses and was actually tested. Hence, conclusions could be drawn based on the theory. However, these studies included other theories as well, which contributed to the theoretical framework together with Rogers’ theory. Table 4 gives an overview of how the theory was used in the studies, assessed using Silva’s classification.

**Table 4:** Classification of theory use based on Silva’s seven criteria

	A purpose of the study is to determine the underlying validity of a theory’s assumptions/propositions	The theory explicitly is stated as the theoretical framework or one of the theoretical frameworks for the research	The theory is discussed in sufficient breadth and depth so that the relationship between the theory and the study hypothesis purposes is clear	The study hypothesis or purposes are deduced clearly from the theory’s assumptions or propositions	The study hypothesis or purposes are empirically tested in an appropriate manner	As a result of this empirical testing, indirect evidence exists of the validity (or lack thereof) of the designated assumptions or propositions of the theory	This evidence is discussed in terms of how it supports, refutes, or explains relevant aspects of the theory	Classification
Bulette Coakley, Barron & Donahue Annese, 2016	No	No	No	No	Unclear	No	No	Minimal use
Lucero & Sousa, 2006	No	Yes	No	No	Yes	Yes	No	Insufficient use
Hurley, 2005	Yes	Yes	Yes	Yes	Yes	Yes	Yes	Adequate use
Rizzo, Gilman & Mersmann, 1994	No	Yes	No	No	No	No	No	Insufficient use
Sherman, 1996	Yes	Yes	Yes	Yes	Yes	Yes	Yes	Adequate use

### 3.3 The quality of the included studies

The studies were found to have varied quality. One study [32] had no quality flaws according to the assessment tool, while the others had varying degrees of quality. This most often concerned lack of identification of and/or dealing with confounders [31, 33] and lack of clarification whether the variables had been measured in a valid and reliable way [30, 33]. One of the studies [33] had more methodological flaws than the others. For example, participant characteristics were insufficiently described. In another study [31], it was unclear whether objective standard criteria were used for measurement of the condition (if the condition was seen as being a nurse). In the mixed-method study [29], there was no rationale for using a mixed-method design. Hence, the contribution of the design to the knowledge field was unclear. Also, the different components of the study did not adhere to the quality criteria of the tradition of each method used, with the procedure and characteristics of participants insufficiently explained. For example, the time between pre- and posttest occasions and the number of participants in the quantitative part were not described. Overviews of the quality assessments are presented in Tables 5, 6.

**Table 5:** Quality appraisal of the quantiative studies using Joanna Briggs Institutes critical appraisal checklist for cross sectional studies [24]

	Were the criteria for inclusion in the sample clearly defined?	Were the study subjects and the setting described in detail?	Was the exposure measured in a valid and reliable way?	Were objective, standard criteria used for measurement of the condition?	Were confounding factors identified?	Were strategies to deal with confounding factors stated?	Were the outcomes measured in a valid and reliable way?	Was appropriate statistical analysis used?
Lucero & Sousa, 2006	Yes	Yes	Unclear	Yes	Yes	Yes	Unclear	Yes
Hurley, 2005	Yes	Yes	Yes	Unclear	Yes	No	No	Yes
Rizzo, Gilman & Mersmann, 1994	Yes	No	Unclear	Yes	No	No	Unclear	Yes
Sherman, 1996	Yes	Yes	Yes	Yes	Yes	Yes	Yes	Yes

**Table 6:** Quality appraisal of the mixed methods study, using Mixed Methods Appraisal Tool (MMAT) [25]

	Is there an adequate rationale for using a mixed methods design to address the research question?	Are the different components of the study effectively integrated to answer the research question?	Are the outputs of the integration of qualitative and quantitative components adequately interpreted?	Are divergences and inconsistencies between quantitative and qualitative results adequately addressed?	Do the different components of the study adhere to the quality criteria of each tradition of the methods involved?
Bulette Coakley, Barron & Donahue Annese, 2016	No	Yes	Yes	Yes	No

### 3.4 Use of the theory-based concept environment in relation to work environment

The deductive qualitative analysis showed that the theory-based concept environment was related to work environment in four of the five included studies [29, 30, 32, 33]. The study that did not include work environment [31] is therefore not represented in this part of this results. The analysis revealed that all aspects of work environment, i.e., human [29, 30, 32, 33], technical and organizational aspects [33] were represented in the studies, but that use of the human aspect was most common. Four of the studies [29, 30, 32, 33] represented the human aspect, but one study represented all three environmental aspect, albeit superficially [33]. The matrix from the deductive analysis is shown in Table 7.

**Table 7:** Matrix over work-environmental aspects in the studies in relation to classification from Oludeyi’s conceptual review [7]

Human (number of studies = 4)	Technical (number of studies = 1)	Organizational (number of studies = 1)
Colleagues giving therapeutic touch to each other in order to decrease work-related stress and heal both the recipient and the provider of the treatment [29].	The physical environment at work, not described in any detail [33].	The organization of the work, which was described as, for example, who is responsible for what [33]
Social support in life, including work, was seen as an environmental part, affecting the human-environment energy field and in turn, manifestations of interpersonal relationships at work [32].
Patients and colleagues are environmental parts to nurses in their working life. For example, that the nurses support each other with work tasks, are in a context of a group culture, and that they struggle to provide high quality care for the patients [33].
Nurses are seen to be in constant interaction with their nursing environment. What this environment consists of is not clearly defined in the article [30], and therefore interpreted in the present study as mainly consisting of other nurses.

### 3.5 Major findings in the studies

One overarching theme emerged in the analysis: *Well-being as an essential phenomenon in working life in all dimensions of existing.* This theme reflects that well-being was a relevant matter for the workers (nurses) as a manifestation all the way from the physical to the existential dimension. Four dimensions were identified in the analysis: The physical, the body-mind, the psychological and the existential. The text below describes the findings in relation to these dimensions. For an overview of the major findings, see Table 4.

*Well-being in relation to the physical dimension* was sparsely described in the studies. However, the study by Bulette Coakley, Barron & Donahue Annese [29] found that therapeutic touch had an impact on physical aspects. Nurses that received the treatment at work had significantly lower respiratory rates after the treatment compared to before the treatment, and one nurse described how his/her back pain eased [29].

All studies addressed aspects of *well-being at the body-mind dimension*. Nurses providing treatments or care experienced/reported improved well-being related to this [29, 33] in terms of comfort and increased energy levels, peacefulness, relaxation and overall well-being.

The nurses also experienced/reported *psychological aspects of well-being*, i.e. focus, stress, anxiety, stimulation, work excitement, job satisfaction, frustration and perceived social support. Nurses experienced increased focus and decreased anxiety when providing therapeutic touch to a colleague [29]. Work excitement were positively correlated with being respected for one’s knowledge, having a stimulating work environment and the opportunity to work with other professionals [33]. A study among nurse managers revealed that they were satisfied with their jobs but anyway scored high levels of stress. Stress was however not related to their job satisfaction but with other factors, such as having direct care responsibilities alongside the manager-work [31]. In contrast, the nurses in Lucero and Sousas study [30] perceived stress only sometimes to fairly often. However, the more perceived stress among the nurses, the less engagement they had in their nursing environment. Stress was also present among nurses that provided therapeutic touch to their colleagues [29]. They could feel that it was difficult to relax during the treatment because they were working at a busy unit and couldn’t stop thinking of everything else they needed to do [29]. The feeling of frustration at work was described among nurses due to low staffing, communication problems, and insufficient time to complete work tasks [33]. Regarding perceived social support, Sherman [32] found that this was positively correlated to nurses’ willingness to care for AIDS patients [32].

**Fig. 2:**
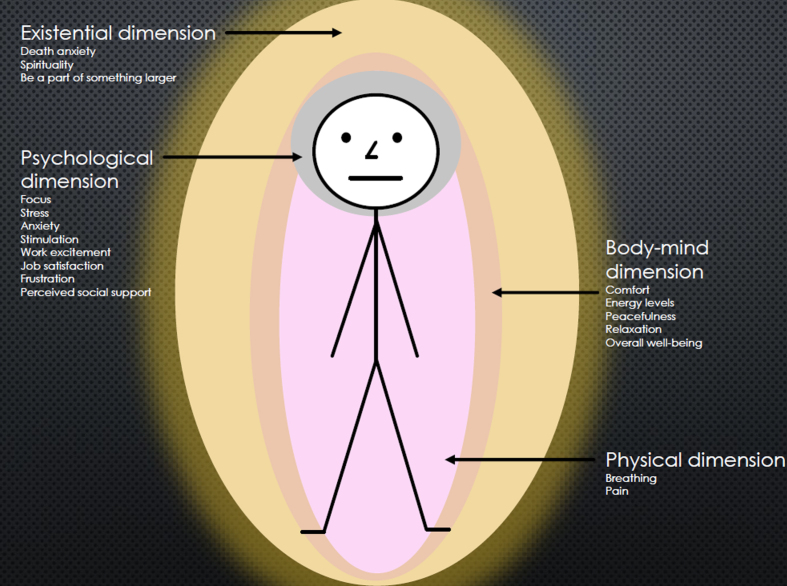
Dimensions of well-being in relation to the worker.

Two of the studies investigated aspects of *well-being at the existential dimension* [29, 32], i.e. being a part of a larger context and experiencing death anxiety and spirituality. Nurses in Bulette Coakley, Barron, and Donahue Anneses study [29] felt that they were a part of, and contributed to, an overall healing environment at work when they engaged in therapeutic touch with their colleagues. In Shermans study [32], the nurses’ death anxiety and spirituality (e.g. sense of a mission in life) were associated with their willingness to care for AIDS patients. In other words, a lower level of death anxiety and higher level of spirituality among the nurses were associated with a higher willingness to care for the patients (and vice versa) [32]. For an overview of the dimensions in relation to each other and to the worker, see Fig. 2.

## 4 Discussion

This literature review showed that Rogers’ science of unitary human beings seems to have been sparsely used in research among workers health and well-being in relation to working life, especially in the last decade, from which only one study was found. All included studies originated from the United States. Most studies had minimal or insufficient use of the theory and the methodological quality varied greatly between the studies. The environment was highlighted in relation to the theory in four of the five included studies, most often regarding other people at work. The major findings concerned various dimensions of well-being and the analysis resulted in one overarching theme: *Well-being as an essential phenomenon in working life in all dimensions of existing.*

Interestingly, there were four quantitative studies and one mixed-method study included in this review. Because of the theory’s high abstraction level, it is difficult to measure the concepts, which may mean that the theory feels more suitable for qualitative research [34]. Four of the five included studies used the theory only minimally or insufficiently, as assessed using Silva’s classification [19]. However, in the present literature review, there was a pattern that those with higher methodological quality also had a more adequate use of the theory, which cannot be assumed [26]. Those studies would be valuable to take as a starting point in future research in the area. It is worth noting that these studies combined Rogers’ theory with other theoretical frameworks to create their hypotheses. Hence, Rogers’ science of unitary human beings was not used adequately on its own. Altogether, this means that the theory has been used only marginally in research among workers health and well-being in working life.

The human aspect, i.e., interaction with other people at work, such as colleagues and patients, was the dominant work environment-related aspect. According to the theory, the environment consists of more than other people – also comprising everything around the individual [5]. Hence, it is reasonable to assume that the theory has the potential to involve more work environment aspects than the human one. According to the World Health Organization [35], all aspects of work environment are important for people’s health and well-being. Since around 2010, we live in the era of “big data,” i.e., in an era of highly developed technology which affects daily life, including work [36]. Rogers’ herself predicted this development in the early 1990s and believed that there was therefore a future need to study how individuals’ humanity can be maintained during increased technological advancement [17]. The lack of technology work environment aspects in this review may be explained by the fact that only one of the included studies in this review was published in the last decade.

Two of the theory’s most central concepts are health and well-being [5]. In the present review, this permeated all major findings more or less explicitly – with different concepts used, such as anxiety, pain and job satisfaction. Martha Rogers made no distinction between the concepts health and well-being, nor did she define them clearly. This is reflected in the research where her theory is used, e.g., this review and a previous review [16]. Both indicate confusion of concepts, or perhaps that the concepts are carelessly used. However, the openness in the relationships between concepts and their definitions can also be viewed as positive, as it means the theory can be used broadly in research related to working life, health, and well-being. The theme that emerged from analysis of the major findings was: *Well-being as an essential phenomenon in working life in all dimensions of existing.* This indicate that well-being among workers, i.e. nurses, is important both at the physical and non-physical dimensions. This supports the theory’s notion of the wholeness of humans [5]. When policies on working life are drawn up and formulated, this pandimensional view on workers should be taken into account in order to promote the health and well-being of workers in the best possible way.

### 4.1 Methodological considerations

This review is subject to some limitations. First, there is a methodological weakness in that the study selection, analyses, and quality assessments were conducted by a single author. This may increase the risk of bias in the results. According to the Cochrane handbook [23] and PRISMA checklist for reporting systematic reviews [22], it is important to minimize the risk of bias. In this current review, there might be some language bias [23], as several studies was excluded in the selection process due to not being in English. Also, two issues of Visions: The Journal of Rogerian Nursing Science were not available, which means that there might be a so-called location bias [23]. These weaknesses were difficult to prevent. However, a specialized librarian helped with the database searches, which is considered as a strength. A large number of search terms within the same concept was used, which is recommended by the Cochrane handbook [23]. However, health, well-being and working life were not searched for in the data bases but instead selected manually to minimize the risk of missing articles due to a too narrow search strategy. Nevertheless, there is always a risk that some relevant articles were not found because concepts from the theory is not visible in title, abstract and key words. This is a known problem regarding nursing theories [19]. The use of only two data bases is also a limitation, even though the two chosen data bases are highly relevant for nursing research [37]. Furthermore, the exclusion of middle-range theories might have biased the results somewhat. For example, Barrett’s theory of power [18] is closely related to Rogers’ science of unitary human beings and some might therefore argue that the present review does not give an accurate picture of the phenomenon under investigation. However, only the original theory was of interest in this review.

### 4.2 Recommendations for future research

The theory has the potential to be used in future research among workers. More aspects of work environment should be targeted – not least technical aspects, considering the contemporary era. I would also suggest more qualitative research as a basis for quantitative research. Research from other parts of the world than the United States would also be beneficial. Furthermore, the theory should be used in research about treatments other than integrative/complementary interventions, for example palliative care and mental healthcare. Because of the theory’s abstract level and that one of its central concepts is an “energy field,” researchers might believe that the theory belongs within “alternative medicine.” However, the essence of the theory is non-controversial and could be used in almost all kinds of nursing research. The theory was used at the individual level in the included studies. This is appropriate, as the theory described individuals and their integrality with the environment. However, there is also a potential to use it at the group level [17] or the organizational level [38]. For example, organizations are in interaction with the surrounding society, which affects their role in society and how well-functioning they are. This might be another way of using Rogers’ theory in relation to work life. The theory could also be used among other professions than nurses, as it is seen as universal and general [5], i.e., not restricted to a certain context or profession. Since we also live in an era in which research about epigenetics [39] is increasing radically, the human-environment interaction is more relevant than ever. A systematic review could be conducted as well, including more data bases and other sources.

## 5 Conclusion

There seems to be a lack of use of Martha Rogers’ science of unitary human beings regarding workers health and well-being in working life, and it has decreased in the last decade. This despite it potentially being more relevant than ever considering, for example, the rapidly evolving technological environment. However, the existing studies reveal the theory’s potential for broad use in the area. It might be time for advancing the research of working life using the science of unitary human beings.

## Ethical considerations

Not applicabale.

## Informed consent

Not applicable.

## Reporting guidelines

The reporting of the study followed the PRISMA-ScR checklist [21].
